# Mixed-methods Study of the Effect of Chemotherapy-induced Peripheral Neuropathy SymptomPermanence on Patient’s Willingness to Alter Neurotoxic Chemotherapy Treatment

**DOI:** 10.21203/rs.3.rs-6304310/v1

**Published:** 2025-05-13

**Authors:** Yerial Jun, Xueting Tao, Jaeyoung Choi, Kelley M. Kidwell, Daniel L. Hertz

**Affiliations:** University of Michigan College of Pharmacy; University of Michigan School of Public Health; University of Michigan College of Pharmacy; University of Michigan School of Public Health; University of Michigan College of Pharmacy

**Keywords:** Chemotherapy-induced peripheral neuropathy, treatment alteration, treatment discontinuation, survey, qualitative research, shared decision making

## Abstract

**Purpose:**

Chemotherapy-induced peripheral neuropathy (CIPN) is a common dose-limiting side effect from taxane and platinum chemotherapy, with symptoms that can persist for years after treatment and significantly diminish quality of life. This study aimed to evaluate how the potential permanence of CIPN influences patient preferences for continuing vs. altering neurotoxic chemotherapy.

**Methods:**

A mixed-methods approach was adopted, which included surveys and semi-structured interviews. During treatment, surveys used the EORTC QLQ-CIPN20 questionnaire to assess CIPN severity and patient preferences for continuing, altering, or discontinuing chemotherapy under hypothetical scenarios of temporary vs. permanent CIPN. Post-treatment interviews investigated patients’ perceptions of altering treatment due to temporary or permanent CIPN.

**Results:**

Survey data from 66 participants revealed that CIPN permanence considerably increased the likelihood of patients preferring to alter treatment (Odds ratio [OR] = 29.14 [95% confidence interval: 15.31–55.46], p < 0.001). Among 29 post-interviewees, 62% decided to continue with their present regimen despite CIPN, citing treatment efficacy and a lack of concern for CIPN. However, in a hypothetical scenario that their CIPN would be permanent, only 8% preferred to continue treatment without alterations, whereas 50% preferred to alter treatment and 13% to discontinue treatment.

**Conclusion:**

CIPN permanence substantially influences patient preferences for treatment decisions. Improved communication between oncology teams and patients regarding risks of permanent CIPN is essential to support shared decision making to achieve patient’s preferred therapeutic outcomes.

## Introduction

National Comprehensive Cancer Network Guidelines recommend treatment with neurotoxic chemotherapy including taxanes and platinums for several tumor types such as breast, colorectal, lung, and ovarian cancer [1, 2]. Although taxane treatment efficacy is well established, treatment is associated with a high rate of chemotherapy-induced peripheral neuropathy (CIPN) [19]. CIPN is characterized by numbness and tingling in the extremities that occurs in more than 50% of taxane-treated patients [16]. CIPN symptoms can improve after treatment but more than half of patients report residual symptoms more than 3 years later[10], including long-term effects on balance and stability [23] and diminished quality of life [3]. The risk of long-term CIPN may outweigh the incremental benefit of continuing taxane treatment, particularly in patients with early-stage breast cancer in whom the risk of long-term recurrence is relatively low.

The only strategy recommended in American Society of Clinical Oncology (ASCO) guidelines for prevention of further CIPN progression in patients with intolerable symptoms is to alter treatment with the offending agent by delaying, decreasing, or discontinuing its administration [14] Up to a quarter of patients receive some treatment alteration due to CIPN [11, 21]. However, prior work indicates that most taxane-treated patients are unaware of the potential long-term effects of CIPN [12] precluding informed discussion with their oncology team regarding the risks and benefits of continued treatment [20]. Including patients in the decision-making process is especially important when it involves an adverse effect that can impact their long-term function and quality of life.

In order to develop tools that can assist in structuring conversations between patients and their medical oncologist, it is critical to understand the patient’s perspective on CIPN and their preferences for being involved in the decision-making process. The purpose of this mixed-methods study was to explore patients’ acceptance of treatment alteration when experiencing CIPN and how the potential permanence of CIPN symptoms affected their preferences.

## Methods

### Study Design and Participants

Data collection was embedded within a prospective observational clinical study conducted for an unrelated purpose. The clinical study enrolled ambulatory patients initiating taxane and/or platinum chemotherapy for breast or colorectal cancer who had access to an iPhone[5]. The study was conducted at the University of Michigan Rogel Cancer Center and approved by the UM IRB-Med (PI: DL Hertz, HUM00171478). All participants completed written informed consent to participate.

### Quantitative Data Collection via Surveys During Treatment

Surveys were completed within an iOS application (NeuroDetect Version 2.0). At the start of cycles 2, 3, and 4 of neurotoxic chemotherapy treatment, patients reported their neuropathy severity via the European Organisation for Research and Treatment of Cancer Quality of Life Questionnaire (EORTC QLQ) CIPN20 patient-reported outcome questionnaire. Each item was rated on a scale from 1 (not at all) to 4 (severe) and the sum score minus 8 (the minimum possible score) was used in the analysis (scale: 0–24, higher numbers indicating more severe CIPN) [18]. Immediately after completing the CIPN assessment, patients were asked about their preferences to continue, alter or discontinue chemotherapy treatment under two hypothetical scenarios: 1) their CIPN symptoms would be temporary or 2) their CIPN symptoms would be permanent.

### Qualitative Data Collection via Interviews After Treatment

Post-treatment interviews were conducted by a study team member (J.C.) to understand patient’s perspectives on altering chemotherapy treatment due to CIPN. A semi-structured interview guide was developed in conjunction with a multidisciplinary research team comprised of experts in clinical-translational CIPN (DH) and outcomes (TS) research (Appendix 1). The interview explored patient’s acceptance of treatment alteration due to CIPN and the effect of CIPN permanence on this decision. Specifically, patients were asked whether they would prefer to continue; delay the next cycle; decrease the dose; or discontinue chemotherapy if they were predicted to experience severe CIPN by the end of treatment. Participants who chose to continue with the chemotherapy without adjustment were then asked the same question under the assumption that their CIPN would be permanent. For both questions, participants’ were asked to explain the reasoning behind their preference.

At the start of each interview, the researcher obtained verbal consent from the individuals to audio record. The audio files were transcribed verbatim except for removal of all identifying information. Interview recruitment was discontinued when thematic saturation was reached.

### Statistical Analysis

The primary outcome of the quantitative analysis was whether patients chose to alter treatment (i.e., delay or discontinue) vs. continue treatment as planned. The main independent variable of interest was CIPN permanence vs. not (i.e., temporary). We analyzed the odds of altering treatment via generalized linear mixed effects models with a random intercept using time as a continuous variable. We additionally controlled for CIPN severity at that time (CIPN20 score), days since start of treatment (continuous), patient’s age (continuous), chemotherapy type (taxane, platinum, or both) and metastatic cancer (vs. non-metastatic) in multivariable analyses.

Interview transcripts were reviewed, and a master codebook was created using Microsoft Excel. Each participants’ responses were coded based on the standardized questions asked during the interview. Response codes were analyzed using descriptive statistics and thematically.

## Results

### Patient Characteristics

A total of 66 patients were enrolled in the study of whom 55 (83%) had breast and 11 (17%) had colorectal cancer (Table 1), and most (82%) had non-metastatic cancer of either type. Neurotoxic chemotherapy regimens were primarily taxane-containing (83%) with or without platinum. The mean age of patients was 50.0 years (Standard Deviation [SD] = 13.3), and the mean duration of chemotherapy treatment was 2.46 months (SD = 1.20).

### Quantitative Effect of CIPN Permanence on Chemotherapy Alteration Preference

**Figure 1:**
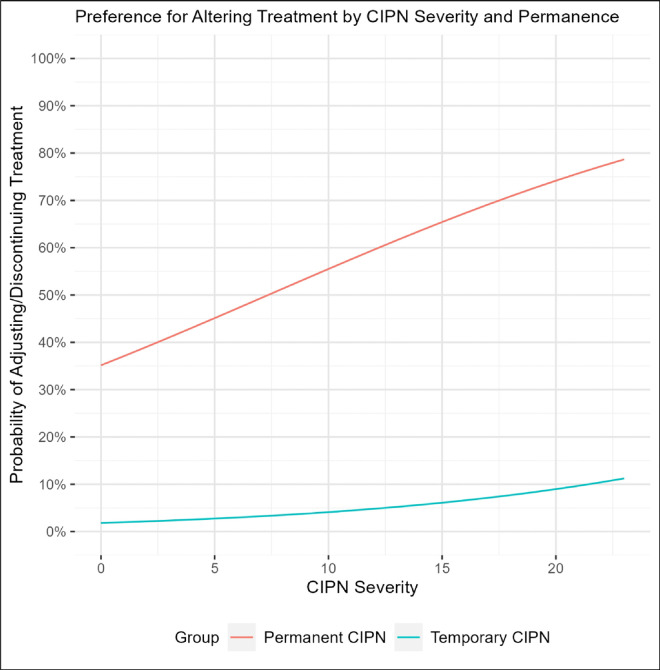
Preference for Altering Chemotherapy Treatment. The relationship between treatment severity (x-axis) and likelihood of preferring to alter (i.e., delay or discontinue treatment [y-axis]), stratified by symptom permanence (permanent [red] vs. temporary [blue]). Preference for altering treatment is much higher when CIPN symptoms are expected to be permanent vs. temporary (Odds ratio: 29.14 (95% confidence interval: 15.31 – 55.46), p<0.001). Other covariates were fixed at their mean or reference value to depict only the three variables of interest (age=50, days since first day of treatment=41, taxane-only treatment, no metastasis).

In the primary analysis, patients were dramatically more likely to choose to alter chemotherapy treatment under the hypothetical scenario that their CIPN symptoms would be permanent compared to when symptoms would be temporary (Odds Ratio [OR] = 29.14 [95% confidence interval: 15.31–55.46], p < 0.001) after adjusting for relevant covariates (Table 2, Fig. 1). In multivariable analyses, CIPN severity, treatment duration, age, type of chemotherapy, and metastases were not associated with the odds of choosing to alter treatment (all p > 0.05).

### Qualitative Analysis of Patient’s Perception on Treatment Alteration

Interviews were conducted September 2021 to September 2022. A total of 29 participants were interviewed (Female = 28, Male = 1). The interviews lasted 6 to 14 minutes with a mean duration of 10 minutes.

When asked about their willingness to alter treatment due to CIPN, 62% (n = 18) of participants wanted to continue with their current regimen (Table 3). The most common themes that emerged regarding why patients wanted to continue treatment was *Prioritizing Cancer Efficacy* (n = 6) and *Lack of Concern Regarding CIPN* (n = 6). No patients indicated they would discontinue treatment due to CIPN but 21% (n = 6) were interested in altering (i.e., decreasing or delaying) dosing and 14% (n = 4) stated they would want to discuss what to do with their provider (see example quotes in Table 3).

Twenty-four participants were then probed with a hypothetical statement that their CIPN would be permanent. Only 8% (n = 2) still wanted to continue treatment as-is, and these participants were highly motivated by *Prioritizing Cancer Treatment Efficacy* (Table 4). Higher percentages of patients wanted to alter treatment (50%, n = 12), discontinue treatment (13%, n = 3), and discuss with their provider (29%, n = 7). Several patients commented that the input from their provider was necessary to make this decision (see example quotes in Table 4).

## Discussion

CIPN is a common dose-limiting side effect that can persist after treatment and irreversibly affect patient’s function and quality of life [3, 10, 23]. Many patients are unaware of the potential long-term impacts of CIPN when considering whether to continue, alter, or discontinue treatment [12, 20]. The primary objective of this mixed-methods study was to evaluate how the permanence of CIPN symptoms influence patients’ preferences for altering chemotherapy during their treatment. We found that the permanence of CIPN symptoms made patients significantly more likely to want to alter chemotherapy and that patients wanted to make this decision collaboratively with their medical oncologist.

This survey of patients currently undergoing neurotoxic chemotherapy found that permanence of CIPN symptoms had an extremely strong influence on their willingness to alter or discontinue chemotherapy (OR ~ 30). Similarly, post-treatment interviews support the effect of CIPN permeance on patient’s preference to alter or discontinue neurotoxic chemotherapy. These findings are consistent with prior research from our group on this topic. A previous survey of patients who had completed neurotoxic chemotherapy treatment found a dramatic decrease in the percentage of patients who would have wanted to continue chemotherapy if their CIPN would be permanent (58–34%) [12]. Additionally, a previous qualitative analysis found that informing patients about the potential for permanent CIPN affects increased their interest in adjusting or discontinuing treatment to preserve quality of life [20].

These interviews also highlighted the importance of shared-decision making when considering whether to alter treatment due to CIPN. Many patients emphasized the need for collaborative discussions with their oncologist to understand the risks of long-term side effects and the potential impact of alteration on treatment efficacy[22]. This supports previous findings that patients are interested in discussing the potential persistence of CIPN symptoms and options for altering treatment with their oncology team [20]. Shared-decision making could be an effective strategy to provide patients with the necessary understanding of the benefits and risks of continuing or altering treatment, so they can make an informed decision that will maximize their likelihood of achieving their personal treatment goals [7]. Currently, there is insufficient data to estimate a patient’s likelihood of experiencing persistent CIPN. The ~ 50% likelihood of CIPN symptoms remaining 3 + years post-treatment has been estimated primarily from large cross-sectional studies of patients many years after treatment or relatively small cohorts of patients followed longitudinally from the end of treatment[10, 15, 17]. Robust data are needed from large prospective cohorts with longitudinal CIPN assessment at the end of and for years post-treatment to understand the actual risk of persistent CIPN [10]. These risk estimates could be further personalized by identifying demographic, treatment, or genetic factors that affect the risk of CIPN persistence [10]. This data could be integrated into decision aids that provide patients with simple information about the risks of persistent CIPN and the potential benefits (reduced persistent CIPN [6]) and risks (reduced efficacy [13]) of treatment alteration [11]. Decision aids have assisted cancer patients and their clinicians with making high-quality shared-decisions in other areas with risk-benefit tradeoffs such as decisions around surgical mastectomy [4, 8, 9].

This study used a patient-centered mixed-methods approach to understand patient preferences around treatment alteration in the context of permanent CIPN. Using actual patients currently receiving treatment enhances the relevance and applicability of these findings whereas the semi-structured interviews allowed for in-depth exploration of patients’ perspectives. There are also limitations of this study that should be considered. The modest sample size, and predominance of women with early-stage breast cancer, limits the reliability of these findings and precludes subgroup analyses to further explore differences between patients or their cancer types or treatment regimens. Additionally, self-reported data can be subject to bias, as patients may provide socially desirable responses.

In conclusion, the permanence of CIPN significantly influences patients’ preferences for altering chemotherapy treatment. There is a critical need for better data to support communication between patients and their oncology teams regarding the potential long-term effects of CIPN, perhaps assisted with decision aids. Prospective trials could then be conducted investigating the effectiveness of these tools to support shared decision-making that improves patient’s likelihood of achieving their goals of chemotherapy treatment.

## Figures and Tables

**Table 1:** Clinical Data for Patients Included in Analysis (n = 66)

		N (%) or Mean (SD)
Age	Years	50.0 (13.3)
Cancer Type	Breast	55 (83.3%)
	Colorectal	11 (16.7%)
Cancer Stage	Non-metastatic	54 (81.8%)
	Metastatic	12 (18.2%)
Chemotherapy Type*	Taxane	55 (83.3%)
	Platinum	23 (34.8%)
Duration of Treatment	Months	2.46 (1.20)

* Some patients received combination treatment with both platinum and taxane

**Table 2:** Odds of Preferring to Alter (Discontinue or Delay) Chemotherapy Treatment vs. Continue Without Alteration

	Odds Ratio (95% Confidence Interval)	P-value
CIPN Symptoms Permanent (vs. Temporary)	29.14 (15.31–55.46)	< 0.001
CIPN Severity (CIPN20 Score)	1.09 (0.99–1.19)	0.07
Duration of Treatment	1.01 (1.00–1.01)	0.18
Age	1.01 (0.95–1.06)	0.84
Platinum-containing Chemotherapy (vs. Taxane)	0.81 (0.11–6.22)	0.84
Platinum-Taxane Combination Chemotherapy (vs. Taxane)	2.98 (0.45–19.8)	0.26
Metastasis (vs. No Metastasis)	0.15 (0.02–1.09)	0.06

**Table 3:** Patient Perceptions on Altering Treatment Based on Evidence of Future CIPN (n = 29)

Continue Treatment	Example Quotes
Prioritizing Cancer Efficacy 31%, N = 9	“I’d rather get the cancer removed and deal later with the possibility of moderate to severe neuropathy” “Because I mean it’s my life. I would want to go with the most aggressive treatment possible”
Lack of Concern about CIPN 21%, N = 6	“I would accept it and continue with treatment”
Finish Treatment on Schedule 10%, N = 3	“[Delaying] just makes the treatment go on longer.”
Alter Treatment	Example Quotes
Prevent Severe CIPN 17%, N = 5	“I would probably want to decrease it if it would prevent [CIPN]”
Appreciate Treatment Pause 7%, N = 2	“it’s nice to have that week of decompression instead of a constant [treatment] every single week.”
Discuss with Provider	Example Quotes
Trust in Provider Expertise 14%, N=4	“I would want to discuss with my doctor … she could lower my dose or [discuss] what we should do.”

**Table 4:** Patient Perceptions on Altering Treatment Based on Evidence of Future CIPN that would be Permanent (n = 24)

Treatment Decision	Example Quote
Continue Treatment 8%, N = 2	“I would want to cure cancer first”
Alter Treatment 50%, N = 12	“If permanent, decrease the dose or listen to the doctor. Stopping wouldn’t be an option unless alternative [treatment was available]”
Discontinue Treatment 13%, N = 3	“I would consider stopping if it was impacting my quality of life and daily activities”
Discuss with Provider 29%, N = 7	“I’m not the one who can make [that] decision” “I honestly believe it’s not really a patient decision. I mean they weigh in, but I don’t know enough about it to decide”
